# Effects of 8 Weeks of Resistance Training Combined with a High-Protein Diet and Omega-3 Supplementation on Body Composition, Muscular Performance, and Muscle-Related Biomarkers in Overweight Women

**DOI:** 10.3390/nu18040611

**Published:** 2026-02-12

**Authors:** Bahareh Radfar, Reza Bagheri, Hamid Ghobadi, Ahmad Hematabadi, Babisan Askari, Amir Rashidlamir, Fred Dutheil

**Affiliations:** 1 Department of Exercise Physiology, Shahrood University of Technology, Shahrood 3619995161, Iran; bahareh.radfar7@gmail.com; 2 Department of Exercise Physiology, University of Isfahan, Isfahan 8174673441, Iran; will.fivb@yahoo.com; 3 Department of Exercise Physiology, Ferdowsi University of Mashhad, Mashhad 9177948951, Iran; ghoba.hamid@gmail.com (H.G.); ahmad.hematabadi@gmail.com (A.H.); 4 Department of Physical Education and Sport Sciences, QaS.C., Islamic Azad University, Qaemshahr 4765161964, Iran; b.askari.1359@iau.ac.ir; 5 Université Clermont Auvergne, CNRS, LaPSCo, Physiological and Psychosocial Stress, CHU Clermont Ferrand, University Hospital of Clermont-Ferrand, Preventive and Occupational Medicine, Witty Fit, F-63000 Clermont-Ferrand, France; fred_dutheil@yahoo.fr

**Keywords:** nutritional supplements, obesity, metabolic health

## Abstract

**Background:** Overweight women are at increased risk of metabolic dysfunction, muscle loss, and reduced physical function during middle age. Resistance training (RT), combined with a high-protein diet and omega-3 supplementation, may help mitigate these risks; however, their combined effects remain unclear. **Objective:** To examine whether omega-3 supplementation enhances the effects of RT combined with a high-protein diet on body composition, muscular performance, and selected biochemical markers in overweight women. **Methods:** Fifty-four overweight women (40–53 years) were randomly assigned to RT plus omega-3 supplementation with a high-protein diet (RO), RT plus placebo with a high-protein diet (RP), or a non-training control group (C). The RT intervention was performed three times per week for 8 weeks. Body composition, muscular performance, and circulating markers related to muscle metabolism and clinical safety were assessed before and after the intervention. **Results:** Forty-four participants completed the study. Both intervention groups demonstrated significant reductions in body mass and fat mass, alongside increases in skeletal muscle mass (SMM) and improvements in muscular strength, endurance, and power compared with the C group (*p* < 0.001). Markers related to muscle metabolism improved in both RT groups, with greater changes observed in the RO group. Clinical safety markers remained within normal ranges, with no between-group differences. **Conclusions:** Eight weeks of RT combined with a high-protein diet effectively improved body composition, muscle function, and anabolic signaling in overweight women. Short-term omega-3 supplementation selectively modulated biochemical markers but did not provide additional improvements in SMM, performance, or clinical safety markers, suggesting that its benefits may be limited without longer-term or higher-dose interventions.

## 1. Introduction

Obesity, characterized by excess fat mass, is associated with increased risk of metabolic and cardiovascular diseases [1,2]. Sex-specific factors such as hormonal fluctuations, menopause, and lifestyle patterns (such as exercise and dietary modification) contribute to differences in adiposity, skeletal muscle mass (SMM), and overall health between women and men [3,4]. Moreover, sedentary behavior in women accelerates this loss, predisposing them to early dynapenia and sarcopenia, conditions that reduce functional capacity and quality of life [5].

Resistance training (RT) is the gold-standard exercise modality for women, as it increases SMM, strength, and body composition [6,7]. Beyond improving lower-limb strength and hypertrophy [7,8], RT helps prevent metabolic and chronic diseases such as diabetes, cardiovascular complications, and fatty liver [9,10,11]. It also mitigates age-related muscle and bone loss, lowering the risk of osteoporosis and sarcopenia in women [12].

In addition to exercise, nutritional strategies play a critical role in optimizing health outcomes. High-protein diets are especially important for women, as protein supports muscle protein synthesis (MPS), facilitates fat mass reduction, and helps preserve SMM during weight management [13,14,15]. Evidence indicates that higher protein intake enhances the adaptive responses to RT, leading to greater improvements in body composition and muscular strength compared with standard protein intake [16]. This synergistic effect of RT and protein ingestion may be particularly beneficial for overweight women, who face elevated risks of muscle loss and metabolic dysfunction.

While some concerns exist regarding potential liver and kidney stress from high-protein diets, most evidence indicates they are safe in healthy individuals [17,18,19,20,21,22]. However, because overweight individuals may have underlying metabolic dysfunction, careful monitoring of biochemical parameters is essential when evaluating high-protein interventions in this population. Beyond protein intake, other nutritional components may further augment the benefits of RT. Among these, omega-3 polyunsaturated fatty acids (n-3 PUFAs) have gained growing attention. Omega-3 fatty acids, considered essential nutrients [23,24], exert multiple health benefits, including attenuation of inflammation, improved cardiometabolic health, and preservation of skeletal muscle [25,26,27]. With aging, women experience a progressive increase in fat mass and decline in SMM, leading to frailty, functional impairment, and cognitive decline [28,29,30]. Omega-3 supplementation, particularly at doses above 2 g/d for at least six months, has been most consistently shown to preserve SMM, strength, and performance [31,32,33]. This temporal requirement suggests that substantial morphological and functional adaptations may be less likely to occur following shorter interventions. However, emerging evidence indicates that shorter-term omega-3 supplementation may still modulate muscle anabolic and catabolic signaling pathways, thereby influencing early metabolic adaptations.

Accordingly, the present study was designed with the realistic hypothesis that, over an 8-week period, omega-3 supplementation combined with RT and a high-protein diet would primarily influence biochemical markers of muscle metabolism, while any additional effects on body composition or muscular performance would be modest relative to the primary adaptations induced by RT and adequate protein intake.

Recent attention has also focused on growth regulators such as myostatin and follistatin, which play key roles in muscle metabolism and obesity. Myostatin, a member of the Transforming Growth Factor-beta (TGF-β) superfamily, inhibits muscle growth, while follistatin, produced primarily in skeletal muscle and liver, acts as a potent myostatin antagonist [34,35,36,37,38,39]. RT downregulates myostatin and upregulates follistatin, thereby promoting muscle hypertrophy [35,40,41]. Given that physical inactivity increases myostatin (particularly) levels and decreases follistatin, these proteins represent important biomarkers for understanding exercise and nutritional interventions in obesity [41,42,43].

While RT and nutritional supplementation independently promote favorable adaptations, there is a notable gap in research examining the combined effects of these strategies during short-term interventions. To our knowledge, no study has explored the interactive influence of RT, a high-protein diet, and omega-3 supplementation over an 8-week period on body composition, muscle performance, and biochemical markers specifically in overweight women. Therefore, this study aimed to investigate whether adding omega-3 supplementation to RT combined with a high-protein diet over an 8-week period provides additional benefits in overweight women. The potential effects of omega-3 in middle-aged women may be particularly relevant because women are generally more resistant to anabolic stimuli than men, suggesting that omega-3 could help augment anabolic responsiveness [44,45,46]. We hypothesized that omega-3 supplementation, when combined with RT and adequate protein intake, would modulate muscle-related metabolic signaling (as reflected by circulating follistatin, myostatin, and their ratio) and contribute to changes in SMM, over an 8-week intervention. Any omega-3–specific effects on SMM were expected to be modest relative to the primary adaptations induced by RT and protein intake.

## 2. Materials and Methods

### 2.1. Participants

Fifty-four overweight women aged 40–53 years were recruited for this study. Participants underwent comprehensive medical screening and were excluded if they had diabetes, hypertension, cardiovascular disease, sleep disorders, or other metabolic conditions. Menopausal status (premenopausal, perimenopausal, or postmenopausal) and hormone replacement therapy (HRT) use were recorded to support later interpretation of results. Eligibility criteria included engagement in less than 1 h of structured exercise per week during the previous year, habitual sleep duration of approximately 7–8 h per night, and no use of dietary supplements or medications, including non-steroidal anti-inflammatory drugs (NSAIDs). Habitual protein intake below 1.6 g·kg^−1^·d^−1^ was verified using dietary recalls prior to enrollment. All eligible participants received a detailed explanation of study procedures and provided written informed consent prior to participation. The study protocol was approved by the Research Ethics Committee of Ferdowsi University of Mashhad (approval code: IR.UM.REC.1403.215) and conducted in accordance with the Declaration of Helsinki.

### 2.2. Study Design

This study was designed as an 8-week randomized, double-blind, placebo-controlled trial. Participants were allocated to one of three groups: resistance training plus omega-3 supplementation with a high-protein diet (RO; *n* = 18), resistance training plus placebo with a high-protein diet (RP; *n* = 18), or a non-training control group (C; *n* = 18). Randomization was performed by an independent researcher using a computer-generated sequence with stratification by body mass index (BMI; <29.0 or ≥29.0 kg·m^−2^). Allocation was concealed using sequentially numbered, opaque, sealed envelopes that were opened only after completion of baseline testing. Participants, outcome assessors, and data analysts were blinded to supplementation assignment (omega-3 vs. placebo). Due to the nature of the intervention, resistance-training supervisors were not blinded to group allocation. Omega-3 capsules (Eurho Vital; 1180 mg fish oil providing 181 mg EPA and 250 mg DHA per capsule, plus 12 IU vitamin E) and placebo capsules (corn oil with 12 IU vitamin E) were identical in appearance, packaging, and labeling to maintain blinding. Supplements were coded by an independent party, and decoding occurred only after completion of data analysis. No unblinding was required due to adverse events. Participants in both training groups received individualized dietary counseling throughout the intervention to achieve a protein intake of approximately 1.6 g·kg^−1^·d^−1^, distributed across 4–6 meals per day while maintaining a slightly positive energy balance. The control group was instructed to maintain their habitual diet and lifestyle. Supplement adherence was monitored using capsule counts and verified by measurement of the plasma omega-3 index in a random subset of participants. Participants were instructed to maintain their usual physical activity outside of the supervised resistance-training sessions and to report any significant lifestyle changes.

### 2.3. Procedures

All participants completed baseline assessments one week prior to the start of the intervention. Measurements included body composition, muscular performance, and biochemical markers, which were conducted on separate days but within a consistent time window (±1 h) to minimize circadian variation. Following baseline testing, participants commenced the 8-week intervention according to group allocation (RO, RP, or C). Supervised resistance-training sessions were performed three times per week on non-consecutive days, with attendance recorded to monitor compliance. Omega-3 or placebo supplementation was consumed daily with meals at consistent times throughout the intervention. Post-intervention assessments were conducted during the week immediately following the final training session and followed the same protocols, order, and time-of-day controls as baseline measurements.

### 2.4. Other Measures

To assess sleep quality and health status, the Pittsburgh Sleep Quality Index (PSQI; range 0–21, higher scores indicate poorer sleep quality) and the General Health Questionnaire-28 (GHQ-28; range 0–84, higher scores indicate worse general health) were used, respectively [47].

### 2.5. Body Composition

Upon arrival at the laboratory, participants were instructed to void completely within 30 min before testing to minimize hydration-related variability. Body mass was measured using a digital scale (Lumbar, Shanghai, China) to the nearest 0.1 kg, and height was measured with a stadiometer (Race Industrialization, China) to the nearest 0.1 cm. BMI, body fat percentage (BFP), and SMM were assessed using a multi-frequency bioelectrical impedance device (Inbody 720, Seoul, South Korea). To control for factors influencing impedance, participants were instructed to fast overnight for at least 12 h, sleep for at least 8 h, and abstain from physical activity and alcohol consumption for 36–48 h prior to testing. The bioelectrical impedance approach has good test–retest reliability (R = 0.96 to 0.98) [48].

### 2.6. Maximal Strength Assessment

Maximal strength was assessed using the one-repetition maximum (1-RM) for both the plate-loaded leg press and chest press. These measurements were also used to prescribe individualized training intensities for the RT protocols. Prior to testing, participants were informed about the purpose of the assessment, potential risks and discomforts, their responsibilities, possible benefits, and their right to withdraw consent at any time. Participants were instructed to abstain from alcohol for 48 h, avoid caffeine for 12 h, and refrain from eating for 2 h before the session, while water intake was permitted.

Testing began with a general 10-min warm-up, consisting of 5 min of light treadmill running (3–5 km/h) or elliptical exercise (levels 5–10), followed by a 5-min specific warm-up that included dynamic exercises such as medicine ball twists (1 × 10), medicine ball wood chops (1 × 10), straddled toe touches (2 × 5), dynamic quadriceps stretches (1 × 5), and medicine ball squats (1 × 5–8). Participants then performed two maximal attempts, and the highest successfully lifted weight and corresponding repetitions were recorded, ensuring that repetitions to fatigue did not exceed 10. Rest periods of 3–5 min were provided between attempts, with no external motivation or verbal encouragement given during testing. Maximal strength was subsequently estimated using the formula: 1-RM = weight/(1.0278 − 0.0278 × repetitions) [49]. The chest press and leg press were selected to represent upper- and lower-body strength, respectively, and the resulting 1-RM values were applied to guide individualized RT prescriptions. All strength assessments were conducted by researchers blinded to group allocation to reduce expectation bias.

### 2.7. Performance Assessment

Physical performance was evaluated using a series of standardized tests targeting muscular strength, endurance, and power. Lower-body functional strength was assessed using the 30-s chair stand test, in which participants were instructed to rise fully from a seated position as many times as possible within 30 s. Core muscular endurance was measured using a plank hold, with participants maintaining the position for the maximum duration possible while keeping proper form. Explosive lower-body power was evaluated via a vertical jump test, in which participants performed a maximal countermovement jump, and jump height was recorded. Upper-body power was assessed using a seated medicine ball throw, with participants projecting a 3 kg medicine ball as far as possible from a seated position.

All tests were demonstrated by the researchers, and participants performed one or two familiarization trials before recording their maximal performance. Standardized instructions and safety precautions were provided throughout testing, and no verbal encouragement or motivational cues were given, consistent with the maximal strength assessments. All performance assessments were conducted by researchers blinded to group allocation to reduce expectation bias. All assessments were conducted on the same day in a standardized order. Participants were given 3–5 min of rest between each test to minimize fatigue and ensure maximal performance. Familiarization trials were completed prior to recording performance to reduce learning effects.

### 2.8. Blood Sampling Analysis

Twenty milliliters of venous blood were collected from the participants’ antecubital vein into plain red-top serum tubes (BD Vacutainer, Franklin Lakes, NJ, USA), which are designed to allow the blood to clot naturally without the use of any anticoagulants or gel separators, in the morning (between 7:00 and 9:00 a.m.) after an overnight fast of at least 10 h. Participants were instructed to refrain from food, caffeine, and strenuous physical activity prior to blood collection. All blood draws were conducted at the same time of day for pre- and post-intervention samples to minimize diurnal variation. Samples were allowed to clot at room temperature for 30 min and then centrifuged at 3000× *g* for 10 min to separate serum from blood cells. Serum was carefully transferred using sterile pipettes, aliquoted into 0.5 mL tubes, and immediately stored at −70 °C until analysis. All assays were performed on the first thaw; no samples underwent more than one freeze–thaw cycle.

Serum concentrations of myostatin (measuring latent/total myostatin, i.e., mostly bound propeptide and latent complex) and follistatin (total human follistatin) were measured in duplicate using commercially available ELISA kits (myostatin: Glory Science Co., Del Rio, TX, USA; follistatin: Glory Science Co). The myostatin assay detection range was 50–3200 pg/mL, with a lower limit of quantification (LLOQ) of 50 pg/mL; the follistatin assay detection range was 0.5–50 ng/mL, with an LLOQ of 0.5 ng/mL. Both assays have been validated for human serum and show minimal cross-reactivity (<1%) with related TGF-β family members. Intra- and inter-assay coefficients of variation were 8.1% and 4.5% for myostatin, and 8.5% and 5.4% for follistatin, respectively.

Additionally, serum liver enzymes, including alanine transaminase (ALT; intra-assay CV: 1.81%, inter-assay CV: 2%), aspartate aminotransferase (AST; intra-assay CV: 2.01%, inter-assay CV: 2.54%), and gamma-glutamyl transferase (GGT; intra-assay CV: 1.56%, inter-assay CV: 0.92%), as well as kidney function markers, creatinine (intra-assay CV: 1.60%, inter-assay CV: 2.24%) and blood urea nitrogen (BUN; intra-assay CV: 2.20%, inter-assay CV: 3.36%), were measured in duplicate using Pars Azmoon kits and the spectrophotometric method (DiaSys Diagnostic Systems GmbH, Holzheim, Germany). All collections were performed at least 48 h after the last RT session to minimize the acute effects of exercise on biochemical markers and to better reflect chronic adaptations.

### 2.9. Resistance Training

Participants completed a supervised, full-body RT program three days per week at 6:00–8:00 p.m. on non-consecutive days, consisting of 9–11 exercises targeting both major and smaller muscle groups using a combination of machines, free weights, and bodyweight exercises. The program included chest press, seated row, leg press, leg curl, leg extension, dumbbell shoulder press, lat pulldown, biceps curl, triceps pushdown, planks for core stabilization, and standing calf raises. Each exercise was performed for 3–4 sets of 8–16 repetitions, with 90 s of rest for larger compound movements and 30–60 s for smaller muscle exercises. Training intensity was prescribed at 50–80% of 1-RM, which was reassessed every 2 weeks to ensure appropriate adjustments were made based on individual progress. The 1-RM was estimated using a standardized method and, to account for participant progression, the load was reassessed and adjusted regularly. Specifically, if a participant completed ≥ 2 repetitions above the upper target for a given exercise in two consecutive sessions, the load was increased by 2.5–5% for subsequent sessions. Training intensity was further monitored and adjusted based on RPE (Rate of Perceived Exertion) and objective load tracking to verify that participants were training within the prescribed intensity range. Adherence to the prescribed sets × repetitions was monitored for each participant, with the percentage of sets completed within the target repetition range recorded as a fidelity metric. Median adherence across exercises was 96% (IQR: 92–99%), indicating high fidelity to the RT prescription. Squats and deadlifts were intentionally excluded from the program to minimize injury risk, as participants were overweight and beginners. All sessions were closely supervised by a qualified exercise specialist to ensure proper technique, safety, and adherence to the prescribed protocol. Table 1 summarizes the RT details.

### 2.10. Diet and Supplementation Protocol

Participants in the RO and RP groups were instructed to maintain a protein intake of approximately 1.6 g·kg^−1^·d^−1^, obtained exclusively from habitual dietary sources, including both plant-based and omnivorous foods, with no additional protein supplements provided. Dietary counseling aimed to maintain participants in energy balance or a slight positive energy balance to support RT adaptations, rather than inducing caloric restriction. At baseline, participants completed six 24-h dietary recalls (four non-consecutive weekdays and two non-consecutive weekend days) to determine habitual protein intake. Throughout the study, participants attended biweekly consultations with an accredited dietitian, who provided guidance on distributing protein intake evenly across 4–6 meals per day, ensuring 20–40 g of protein per meal to optimize MPS. Total daily energy intake and macronutrient distribution were monitored throughout the intervention using daily food records; however, energy expenditure was not directly measured, and therefore energy balance was inferred from reported intake rather than formally verified. Daily food records were maintained using mobile applications (Easy Diet Diary for iOS and MyFitnessPal for Android), and all dietary data were analyzed using a standardized database (Diet Analysis Plus, version 10; Cengage) to ensure consistency across participants.

The C group did not receive dietary counseling and was asked to maintain their habitual diet and lifestyle throughout the intervention. The protein intake of ~1.6 g·kg^−1^·d^−1^ was selected based on evidence indicating this amount supports maximal fat-free mass (FFM) gains in response to RT. Omega-3 supplementation was administered at a total daily dose of 3 g (three 1-g capsules). Participants were instructed to take all capsules with their main meal to optimize fat-soluble absorption; those experiencing mild gastrointestinal discomfort were permitted to divide the dose across two or three meals.

### 2.11. Statistical Analysis

The primary outcome was the change in SMM. The sample size was determined using G*Power version 3.1.9.2 for a within-between interaction (3 groups × 2 time points) in repeated measures ANOVA, with SMM as the endpoint. An effect size of 0.25, statistical power of 80%, and α = 0.05 indicated that 42 participants (14 per group) were required. To account for an anticipated 20% dropout rate, we enrolled 54 participants (18 per group). Analyses were conducted on participants who completed both baseline and post-intervention assessments (completers analysis). A two-way repeated measures ANOVA (group × time) was used to evaluate differences in the primary outcome (SMM) and secondary outcomes (muscle performance and biochemical markers). Sphericity was evaluated using Mauchly’s test, and in cases of violation, the Greenhouse-Geisser correction was applied to adjust the degrees of freedom. Post hoc pairwise comparisons were performed with Bonferroni correction to adjust for multiple testing. Normality of residuals was assessed using the Shapiro–Wilk test. Baseline differences between groups were assessed using one-way ANOVA. Given that SMM was a primary outcome, Pearson correlation analyses were conducted to explore its associations with changes in other outcome variables, providing insight into potential mechanistic links between the intervention and observed adaptations. These analyses were considered exploratory to provide insight into potential relationships and were interpreted cautiously to avoid overgeneralization. Differences in categorical variables, including menopausal status and HRT use, were assessed using Fisher’s exact test. Figures were generated using GraphPad Prism (version 8.4.3, 686). Statistical significance was set at *p* < 0.05.

## 3. Results

### 3.1. Primary Endpoint and Multiplicity Control

The primary outcome was SMM, while other body composition indices, performance measures, and biochemical markers were considered secondary outcomes. All pairwise comparisons were Bonferroni-adjusted to account for multiple testing.

### 3.2. Menopausal Status and HRT Use

Menopausal status and HRT use were recorded for all participants and are summarized in Table 2. Fisher’s exact test indicated no significant differences among the groups across the menopause periods (*p* > 0.072). The findings indicated no significant difference between the groups with respect to HRT (*p* > 1.00).

### 3.3. Body Composition

There were significant time × group interactions for body mass (F = 18.88; *p* < 0.001), BMI (F = 19.08; *p* < 0.001), BFP (F = 24.42; *p* < 0.001), and SMM (F = 16.34; *p* < 0.001). Body mass significantly decreased in both the RO and RP groups. In RO, the reduction was Δ −3.01 kg (95% CI = −3.99 to −2.02; *p* < 0.001), and in RP it was Δ −1.31 kg (95% CI = −1.75 to −0.88; *p* < 0.001). BMI significantly decreased in RO (Δ −1.20 kg.m^−2^; 95% CI = −1.59 to −0.81; *p* < 0.001) and RP (Δ −0.53 kg.m^−2^; 95% CI = −0.70 to −0.35; *p* < 0.001). BFP significantly decreased in RO (Δ −3.45%; 95% CI = −4.76 to −2.14; *p* < 0.001) and RP (Δ −2%; 95% CI = −2.37 to −1.69; *p* < 0.001). Conversely, SMM significantly increased in RO (SMM: Δ 0.75 kg; 95% CI = 0.39 to 1.10; *p* < 0.001) and RP (SMM: Δ 0.77 kg; 95% CI = 0.53 to 1.01; *p* < 0.001). No significant changes were observed in the C group, and no between-group differences were detected between RO and RP for any body composition variable (all *p* > 0.05).

### 3.4. Performance

There were significant time × group interactions for chest press (F = 54.04; *p* < 0.001), leg press (F = 94.87; *p* < 0.001), chair stand (F = 77.87; *p* < 0.001), plank hold (F = 80.87; *p* < 0.001), vertical jump (F = 44.50; *p* < 0.001), and medicine ball throw (F = 70.56; *p* < 0.001). Upper- and lower-body strength (chest and leg press), muscular endurance (chair stand and plank), power (vertical jump), and upper-body explosive strength (medicine ball throw) improved from pre- to post-intervention in both RO and RP groups. In RO, improvements were chest press = Δ 5.30 kg (95% CI = 4.34–6.27), leg press = Δ 9.48 kg (95% CI = 8.17–10.80), chair stand = Δ 6.87 repetitions (95% CI = 6.03–7.70), plank hold = Δ 5.98 s (95% CI = 5.02–6.94), vertical jump = Δ 3.56 cm (95% CI = 3.04–4.09), and medicine ball throw = Δ 0.53 m (95% CI = 0.46–0.59). In RP, chest press = Δ 4.41 kg (95% CI = 3.49–5.34), leg press = Δ 11.27 kg (95% CI = 9.38–13.16), chair stand = Δ 7.36 repetitions (95% CI = 6.49–8.22), plank = Δ 6.71 s (95% CI = 5.78–7.64), vertical jump = Δ 2.74 cm (95% CI = 2.24–3.24), and medicine ball throw = Δ 0.68 m (95% CI = 0.55–0.82). The C group showed no significant changes (*p* > 0.05), and no differences were observed between RO and RP groups for any variable (*p* > 0.05).

### 3.5. Biochemical Markers

#### 3.5.1. Muscle-Related Markers

There was significant time × group interactions for follistatin (F = 49.28; *p* < 0.001), myostatin (F = 272.46; *p* < 0.001), and follistatin-to-myostatin ratio (F = 86.48; *p* < 0.001). Follistatin significantly increased in RO = Δ 0.52 ng/mL (95% CI = 0.42 to 0.62; *p* < 0.001) and RP = Δ 0.33 ng/mL (95% CI = 0.27 to 0.39; *p* < 0.001), with no significant change in the C group (*p* = 0.737). Myostatin levels significantly decreased in RO = Δ −2.89 ng/mL (95% CI = −3.09 to −2.69; *p* < 0.001) and RP = Δ −2.04 ng/mL (95% CI = −2.18 to −1.89; *p* < 0.001), with no significant change in C (*p* = 0.950). Consequently, the follistatin-to-myostatin ratio increased significantly in RO = Δ 0.23 (95% CI = 0.19 to 0.27; *p* < 0.001) and RP = Δ 0.10 (95% CI = 0.09 to 0.12; *p* < 0.001), while the ratio remained unchanged in the C group (*p* = 0.761). Increases in follistatin, decreases in myostatin, and increases in their ratio were significantly greater in RO compared to both RP and C.

#### 3.5.2. Kidney and Liver Function

There were significant time × group interactions for ALT (F = 49.99; *p* < 0.001), AST (F = 25.51; *p* < 0.001), GGT (F = 45.02; *p* < 0.001), creatinine (F = 29.11; *p* < 0.001), and BUN (F = 61.48; *p* < 0.001). Significant increases were observed in liver enzymes and kidney markers in both RO and RP groups. In RO, ALT increased (Δ = 2.74 U/L; 95% CI = 2.28 to 3.20; *p* < 0.001), AST (Δ = 2.94 U/L; 95% CI = 2.30 to 3.59; *p* < 0.001), and GGT (Δ = 3.69 U/L; 95% CI = 3.00 to 4.39; *p* < 0.001). Similarly, in RP, ALT increased (Δ = 2.41 U/L; 95% CI = 2.03 to 2.80; *p* < 0.001), AST (Δ = 3.67 U/L; 95% CI = 2.85 to 4.50; *p* < 0.001), and GGT (Δ = 3.82 U/L; 95% CI = 2.93 to 4.71; *p* < 0.001). No significant changes were observed in the C group for ALT, AST, or GGT (*p* > 0.05).

For kidney function, creatinine significantly increased in RO (Δ 0.15 mg/dL; 95% CI = 0.11 to 0.19; *p* < 0.001) and RP (Δ 0.11 mg/dL; 95% CI = 0.08 to 0.15; *p* < 0.001). BUN also significantly increased in RO (Δ 1.18 mg/dL; 95% CI = 0.97 to 1.40; *p* < 0.001) and RP (Δ 1.31 mg/dL; 95% CI = 1.11 to 1.51; *p* < 0.001). No significant changes were observed in the C group for creatinine or BUN (*p* > 0.05). There were no significant differences for all markers between RO and RP (*p* > 0.05).

### 3.6. Correlations Between SMM, Biochemical Markers, and Performance

**Figure 1:**
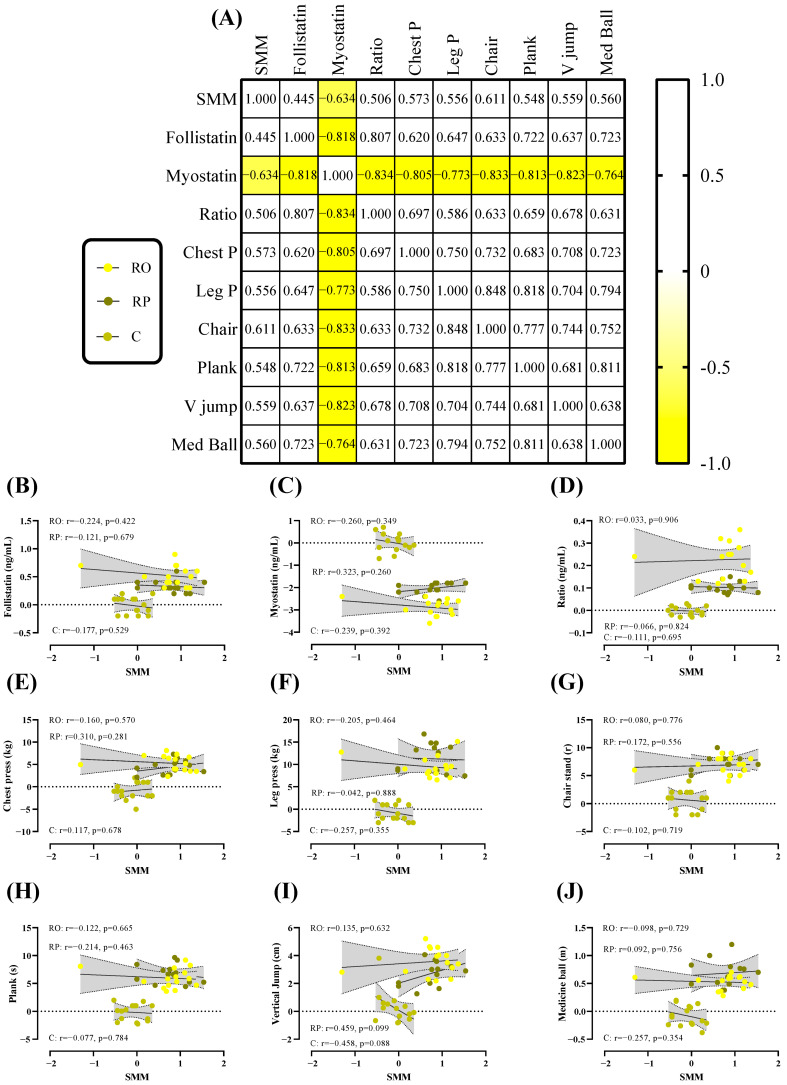
Relationships between change in SMM (Δ SMM (kg)) and change in biochemical and performance variables (Δ (variable)). (**A**) Correlation matrix of Δ SMM and biochemical and performance variables; r values as shown. Key indicates magnitude of r (yellow = −1, white = 1). (**B**–**J**) linear regression (Pearson’s) of Δ (variable) as a function of Δ SMM (kg). Linear regression indicated by solid black line; 95% confidence intervals indicated by gray zones.

To explore potential relationships between training-induced changes in SMM (ΔSMM) and changes in biochemical and performance variables (Δ [variable], irrespective of group: RO, RP, or C; Figure 1), a correlation matrix was first generated. Most variables exhibited moderate positive associations with ΔSMM, whereas myostatin showed a strong negative relationship. Linear regression analyses of individual Δ [variable] as a function of ΔSMM were evaluated using an extra sum-of-squares F test to determine whether pooled data could be modeled as a single condition; however, no dataset met this criterion. When analyzed by group, the RO showed no significant correlations between ΔSMM and any performance variables, including chest press, leg press, chair stand, plank, vertical jump, or medicine ball throw (all *p* > 0.340). In the RP, ΔSMM displayed a positive, though non-significant, correlation with vertical jump height (r = 0.459, *p* = 0.099), while no other performance measures were associated with ΔSMM (all *p* > 0.260). Overall, gains in SMM were not consistently linked to improvements in performance and biochemical markers across groups.

### 3.7. Adherence and Compliance

Adherence to both the training and dietary interventions was high. Median training session attendance was 98% (IQR: 95–100%) across groups. Attendance was defined as completion of ≥85% of scheduled sessions. If a session was missed, a make-up session was arranged within the same week and counted toward adherence; with make-ups included, all participants completed the prescribed total number of sessions. Exercise load and intensity were supervised and progressed according to the program, with RPE monitored at each session. Dietary compliance was defined as achieving the prescribed protein intake (1.5–1.7 g·kg^−1^·d^−1^) on at least 80% of reporting days. Median compliance was 92% (IQR: 88–96%). No participants reported gastrointestinal discomfort or other adverse events.

### 3.8. Participant Characteristics

**Figure 2:**
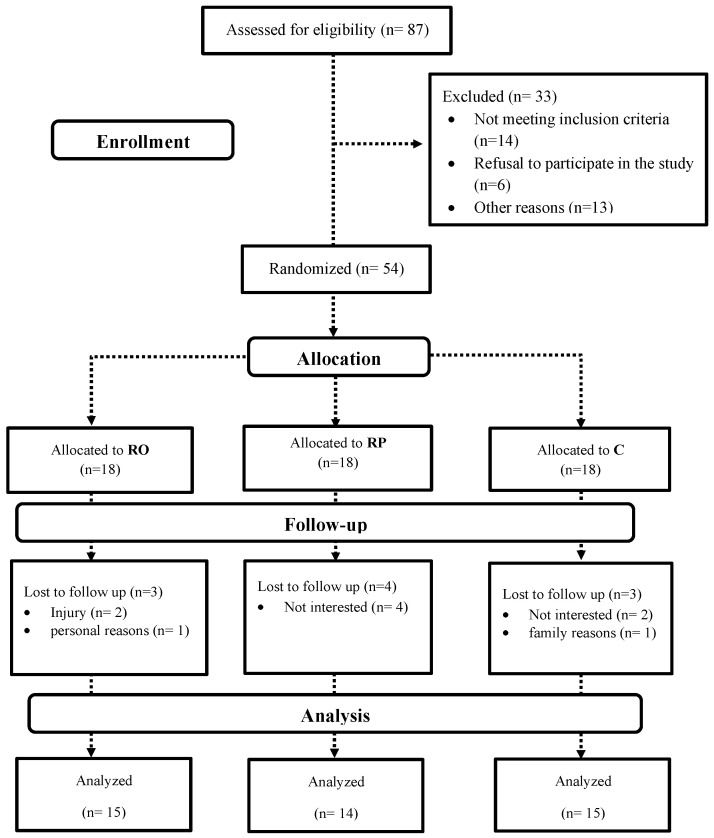
Flowchart of Participants. RO, resistance training + 1.6 g·kg^−1^·d^−1^ + omega 3; RP, resistance training + 1.6 g·kg^−1^·d^−1^ + placebo; C, control.

Of the 54 participants initially enrolled, 10 withdrew (RO = 3; RP = 4; C = 3) due to personal reasons, lack of time, or loss of interest. The remaining 44 participants (RO = 15; RP = 14; C = 15) successfully completed the training and dietary interventions (Figure 2). There were no differences between groups for PSQI (*p* = 0.578) or GHQ-28 (*p* = 0.616), and no significant differences in any baseline characteristics, except for BFP (Table 2).

### 3.9. Dietary Assessments

Dietary intakes are shown in Table 3. In RO, energy intake increased (Δ = 3.38 kcal·kg^−1^·d^−1^; 95% CI = 1.34 to 5.43; *p* = 0.003), corresponding to an absolute increase (Δ = 167.44 kcal·d^−1^; 95% CI = 6.05 to 328.84; *p* = 0.043), while protein intake increased (Δ = 0.96 g·kg^−1^·d^−1^; 95% CI = 0.92 to 1.00; *p* < 0.001). No significant changes were observed for fat (*p* = 0.737) or carbohydrate (*p* = 0.597). In RP, energy intake increased (Δ = 4.41 kcal·kg^−1^·d^−1^; 95% CI = 2.09 to 6.73; *p* = 0.001), corresponding to an absolute increase (Δ = 263.70 kcal·d^−1^; 95% CI = 105.66 to 421.73; *p* = 0.003), while protein intake increased (Δ = 1.01 g·kg^−1^·d^−1^; 95% CI = 0.96 to 1.05; *p* < 0.001). No significant changes were found for fat (*p* = 0.972) or carbohydrate (*p* = 0.530). Energy and protein intake did not differ between RO and RP. In C, no significant changes were observed in energy, macronutrient, or protein intake (all *p* > 0.34).

## 4. Discussion

This study investigated the combined effects of RT and 3 g/day of omega-3 supplementation in overweight women. Compared with the C group, both intervention groups demonstrated significant improvements in anthropometric variables, including body mass, BFP, and SMM. Reductions in BFP were comparable between the training groups. Both interventions also resulted in substantial gains in muscular strength, endurance, and power relative to the C group. Notably, both training groups exhibited increased serum follistatin and decreased myostatin concentrations, leading to a marked elevation in the follistatin-to-myostatin ratio, which was significantly greater in the RO compared with the RP group. In addition, liver enzyme levels were higher in both intervention groups compared with C, while creatinine and blood urea nitrogen concentrations were significantly elevated in both training groups. Contrary to our primary hypothesis, omega-3 supplementation did not confer additional increases in SMM or performance beyond RT combined with a high-protein diet over the eight-week intervention. Importantly, the short-term increases in SMM observed in both training groups likely reflect the well-established effects of RT combined with adequate protein intake on SMM, whereas omega-3–specific effects on detectable changes in SMM may require longer supplementation periods. These findings suggest that, within a short-term intervention, omega-3 fatty acids may preferentially influence anabolic–catabolic signaling pathways rather than translate into additional measurable changes in SMM or functional performance.

### 4.1. Body Composition

Alterations in body composition during middle age are closely linked to hormonal fluctuations, a reduction in basal metabolic rate, and lower levels of physical activity [50,51]. RT plays a significant role in enhancing SMM, physical strength, and body composition in middle-aged women [52,53]. Evidence consistently supports RT as an effective intervention for reducing fat mass and increasing lean mass. For example, a 12-week RT program led to reductions in fat mass in middle-aged women with obesity [54], and Da Silva et al. (2023) reported that higher RT loads are essential for improving body composition in postmenopausal women [55]. A meta-analysis further confirmed that RT improves body composition in individuals classified as overweight or obese [56]. Our study’s findings align with these reports, demonstrating that eight weeks of RT combined with either omega-3 or placebo supplementation, alongside a high-protein diet, led to significant reductions in body mass and BFP, and increases in SMM in both intervention groups (RO and RP). No significant changes were observed in the C group, confirming that the improvements in body composition were due to the interventions. RT increases muscle cells by mitigating muscle proteolysis, leading to an elevation in basal metabolic rate [57]. Additionally, RT-driven hormonal and myokine changes enhance fat oxidation, supporting fat reduction [58,59,60].

Participants in both intervention groups consumed approximately 1.6 g·kg^−1^·d^−1^ of protein, a level recognized as optimal for maximizing MPS and promoting favorable body composition changes when combined with RT [21,61,62]. This represented an increase in total energy intake of ~3–4 kcal·kg^−1^·d^−1^, primarily from protein rather than carbohydrates or fats, which may have contributed to the observed improvements in SMM and body composition. Since participants’ baseline protein intake was lower and total energy intake remained stable, the increase in dietary protein and initiation of RT likely created an anabolic environment sufficient to improve SMM and reduce BFP. Consequently, adequate protein intake [63,64] may have minimized any additional anabolic effects of omega-3 supplementation. It is important to note that the structured and supervised supplementation protocol implemented in this trial contrasts with the largely self-prescribed and heterogeneous dietary supplement use commonly reported in fitness populations [65], reinforcing the need for evidence-based guidance when integrating nutritional supplements into training programs. Similar findings were reported by Cornish et al. (2018), where 12 weeks of omega-3 (EPA and DHA) combined with RT produced no additional improvement in body composition versus RT alone [66], and by Félix-Soriano et al. (2021), who observed no significant body composition changes after 16 weeks of RT and omega-3 supplementation [67]. These results align with the current study and contrast with some reports suggesting synergistic effects between RT and omega-3 on body composition [68,69].

The proposed anabolic actions of omega-3 fatty acids include reducing inflammation, attenuating catabolic signaling, and enhancing muscle protein synthesis sensitivity. Omega-3s may suppress TNF-α and IL-6 activity, reducing NF-κB-mediated protein breakdown while promoting anabolic signaling [27,70,71]. Additionally, EPA and DHA may upregulate PGC-1α and PPARs, facilitating mitochondrial biogenesis and increasing fatty acid oxidation [72,73]. Therefore, the absence of additional omega-3 benefits in this study may be attributed to the short intervention period, sufficient protein intake, and the presence of anabolic resistance often observed in middle-aged women.

### 4.2. Performance

Both intervention groups demonstrated notable improvements in strength, endurance, and muscular power compared with the C group, with no significant differences between the two intervention groups. Numerous studies have shown that RT effectively enhances muscle performance [9,74,75]. For instance, Benton et al. (2011) found that eight weeks of RT improved muscular strength in previously untrained middle-aged women [53]. Similarly, Holviala et al. (2006) reported that a structured strength-training program significantly increased maximal and explosive leg-extensor strength, walking speed, and dynamic balance [76]. Candow and Burke (2007) observed comparable strength gains when previously untrained adults performed volume-matched RT either two or three times per week over six weeks [77]. The primary mechanism driving early strength gains from RT is neuromuscular adaptation characterized by improved muscle activation, higher motor unit firing rates, better coordination, and optimized agonist–antagonist muscle activation [78,79]. Consistent with previous work, the present findings show that eight weeks of RT significantly enhanced muscle strength in middle-aged women. Increased muscular strength in turn positively influences both endurance and power, since strength, a measure of maximal force generation, underpins the capacity for repeated contractions (endurance) and rapid force development (power) [80]. Participants in both intervention groups consumed approximately 1.6 g·kg^−1^·d^−1^ of protein, an intake considered sufficient to support optimal neuromuscular adaptations to RT. Before the intervention, protein intake was below this threshold, so the observed performance improvements likely stem from both the training stimulus and increased protein consumption. However, despite the observed increase in the follistatin-to-myostatin ratio in the RO group, the addition of omega-3 supplementation did not further enhance strength, endurance, or power. This underscores the complexity of translating systemic molecular changes into functional adaptations, particularly in short-term interventions. This contrasts with some studies reporting performance benefits from omega-3 supplementation, a discrepancy that may be explained by differences in supplementation dose, duration, or participant characteristics. For example, Okut et al. (2025) found that eight weeks of RT combined with 3150 mg·day^−1^ of omega-3 (EPA and DHA) significantly improved strength, power, agility, and reaction time compared with C [81]. These benefits have been linked to omega-3–induced changes in muscle cell membrane phospholipids, enhancing membrane fluidity, permeability, and signaling efficiency [82,83]. Improved membrane properties may facilitate nutrient transport, hormonal signaling, and contractile efficiency. Additionally, omega-3 supplementation can enhance mitochondrial function and ATP production efficiency, supporting endurance performance [84,85].

### 4.3. Muscle-Related Markers

Follistatin and myostatin are key regulatory proteins that play crucial roles in muscle growth and development. The interaction and balance between these two factors have drawn significant attention in physiology, sports science, and medical research due to their relevance to hypertrophy, the process of muscle enlargement [86,87,88]. The present findings revealed that follistatin levels increased and myostatin levels decreased similarly in both training groups; however, the follistatin-to-myostatin ratio was significantly higher in the RO group compared with the RP group. Participants in both groups consumed approximately 1.6 g·kg^−1^·d^−1^ of protein, likely providing sufficient substrate to promote MPS and amplify the adaptive effects of RT, thereby supporting changes in follistatin and myostatin levels.

Previous research consistently shows that RT elevates follistatin and reduces myostatin, although some studies report conflicting results [49,89,90,91]. Myostatin is present both locally within skeletal muscle and systemically in plasma. The localized pool is released following sarcomere or sarcolemma disruption and plays a role in the muscle’s adaptive protein synthesis response to eccentric contractions [92]. A meta-analysis confirmed that RT significantly lowers myostatin while increasing follistatin, promoting muscle growth by reducing myostatin’s inhibitory effects [88]. Mechanistically, follistatin acts as a potent antagonist of myostatin by binding to and neutralizing its biological activity, thereby preventing activation of Smad signaling pathways. This fosters an anabolic environment that enhances muscle regeneration and hypertrophy [93,94]. The role of omega-3 supplementation in modulating follistatin and myostatin remains less clear. It has been proposed that omega-3s may attenuate myostatin activity through their anti-inflammatory and antioxidant effects, reducing inflammation and oxidative stress [95,96]. Moreover, omega-3s may support follistatin-mediated activation of the mTOR pathway, promoting an anabolic state conducive to muscle growth [97,98]. The greater increase in the follistatin-to-myostatin ratio in the RO group suggests that omega-3 supplementation may augment the anabolic environment induced by RT, potentially through anti-inflammatory mechanisms, suppression of NF-κB signaling, and upregulation of PGC-1α, particularly when combined with adequate protein intake to maximize MPS. However, changes in circulating follistatin and myostatin may not directly reflect muscle-specific or intracellular signaling events, which may partly explain the absence of corresponding differences in SMM or performance outcomes over the eight-week intervention. Accordingly, while the enhanced follistatin-to-myostatin ratio in the RO group may reflect a more favorable systemic anabolic milieu, such circulating biochemical adaptations may represent early or preparatory responses that do not necessarily translate into detectable changes in SMM or functional performance over a short-term intervention.

### 4.4. Kidney and Liver Function

In the present study, ALT, AST, GGT, creatinine, and BUN increased significantly in both intervention groups compared with the control, with no significant differences between RO and RP. These changes were mild, and all values remained within clinically accepted ranges (ALT: 7–56 U/L; AST: 10–40 U/L; GGT: 9–48 U/L; creatinine: 0.6–1.2 mg/dL; BUN: 7–20 mg/dL). Such elevations are consistent with prior reports showing that RT and high-protein diets (~1.6 g·kg^−1^·d^−1^) can transiently increase creatinine, BUN, and transaminases without indicating organ damage [21,99], likely reflecting normal metabolic adaptations rather than pathological responses. Although prolonged excessive protein intake has been suggested to contribute to glomerular damage, this effect appears minimal in individuals with healthy kidneys [22,100]. Similarly, short-term elevations in liver and kidney markers observed with higher protein intakes do not necessarily indicate clinically relevant acute organ injury over an eight-week period [21]. Beyond protein-related adaptations, omega-3 fatty acids are reported to have hepatoprotective and renoprotective effects. Experimental and clinical evidence suggests omega-3 can attenuate APAP-induced toxicity via Nrf2-mediated antioxidant and NF-κB-related inflammatory pathways [101], and improve liver function, insulin resistance, and lipid metabolism in NAFLD [102,103,104]. Omega-3 may also support renal function in CKD through anti-inflammatory mechanisms [105]. However, in our study, omega-3 supplementation did not attenuate the liver or kidney marker increases, likely because participants were otherwise healthy, the intervention was short-term, and the protein intake and RT stimulus predominated.

This discrepancy may reflect sensitivity to dose or duration, the overriding effect of high-protein intake combined with RT on nitrogen metabolism, or the limited timeframe of the intervention. Future studies should explore longer interventions, follow-up assessments, graded protein targets, and dose–response omega-3 regimens to better understand these interactions. It is important to distinguish statistical significance from clinical relevance when interpreting these findings. Although liver and kidney markers increased significantly in the intervention groups, all values remained within established clinical reference ranges, suggesting no evidence of clinically meaningful hepatic or renal dysfunction over the eight-week intervention. Nevertheless, the present findings should not be interpreted as evidence of long-term safety, as the relatively short duration of the study precludes conclusions regarding the chronic effects of RT combined with high protein intake and omega-3 supplementation on liver and kidney function.

### 4.5. Correlations

Correlations between SMM, biochemical markers, and performance were generally inconsistent and, at times, difficult to interpret. In the RO group, no significant associations were observed between ΔSMM and any performance variable, including chest press, leg press, chair stand, plank, vertical jump, or medicine ball throw (all *p* > 0.340). In the RP group, ΔSMM showed a positive but non-significant correlation with vertical jump height (r = 0.459, *p* = 0.099), while all other performance measures remained uncorrelated (all *p* > 0.260). Taken together, these exploratory analyses indicate that gains in total SMM were not consistently linked to improvements in performance. One likely explanation is that total body SMM may not capture regional adaptations that more directly influence task-specific performance. For example, increases in lower body SMM may be more relevant for leg press or vertical jump performance, while upper body hypertrophy may better predict chest press or medicine ball throw outcomes. Additionally, variability in neuromuscular adaptations, motor learning, and technique improvements could further decouple changes in SMM from functional performance over an eight-week intervention. Future studies should consider assessing regional SMM changes and muscle quality alongside task-specific performance to clarify these relationships.

### 4.6. Limitations

Several limitations should be considered when interpreting the findings of this study. Most importantly, body composition was assessed using bioelectrical impedance analysis (BIA) rather than dual-energy X-ray absorptiometry (DXA). Although BIA is a reliable and widely used method, it is inherently less precise than DXA. This is particularly relevant given that the observed increases in SMM (approximately 0.75–0.77 kg) were modest and may approach the measurement error associated with BIA, despite its reported good test–retest reliability. In addition, while hydration status was partially standardized through pre-test instructions (voiding, fasting, sleep, physical activity, and alcohol abstinence), BIA remains more sensitive to hydration fluctuations than DXA, which may have influenced the estimation of SMM changes.

Another key limitation related to the study aim is that myostatin and follistatin were assessed in serum rather than within skeletal muscle tissue. Circulating concentrations may not fully reflect intracellular signaling or local muscle-specific regulation. For example, despite a greater follistatin-to-myostatin ratio in the RO group compared with the RP group, gains in total SMM were similar between groups. Direct assessment of intramuscular myostatin and follistatin expression would likely provide a more accurate insight into the anabolic mechanisms underlying the observed adaptations. The study also focused on short-term responses; therefore, the long-term effects of the interventions on liver, kidney, and metabolic health remain unclear and warrant further investigation. In addition, fixed doses of omega-3 supplementation and a standardized high-protein diet were employed. Variations in dosage, timing, or distribution of these dietary strategies may lead to different physiological responses and should be explored in future studies. Although menopausal status and hormone replacement therapy use were recorded and did not differ between groups, the study was not powered to examine outcomes according to menopausal stage or hormone therapy, which may influence muscle and body composition responses.

Furthermore, the observed improvements may be largely attributable to the RT stimulus itself, as RT is a well-established and potent intervention for increasing SMM and function. While this study examined the combined effects of RT, a high-protein diet, and omega-3 supplementation, future research should aim to disentangle the independent and interactive contributions of each component. The absence of inflammatory marker assessments also limits interpretation, as such measures could have provided additional insight into the potential anti-inflammatory mechanisms of omega-3 supplementation in overweight women.

Additional methodological considerations include the reliance on self-reported dietary intake, which may be subject to under- or over-reporting despite efforts to monitor adherence. Although the plasma omega-3 index was measured in a random subset of participants to verify compliance, red blood cell omega-3 content was not assessed in the full cohort. A more comprehensive assessment of omega-3 status would strengthen conclusions regarding physiological incorporation and adherence. Finally, analyses were conducted using a completers-only approach, which may introduce bias and reduce statistical power, particularly given the approximately 19% dropout rate for some secondary outcomes.

## 5. Conclusions

Eight weeks of RT combined with a high-protein diet effectively enhanced SMM, strength, and anabolic signaling in overweight women. Short-term omega-3 supplementation selectively altered biochemical markers, such as the follistatin-to-myostatin ratio, but did not translate into additional improvements in SMM, functional performance, or clinical safety markers. These findings suggest that, in the context of adequate protein intake and RT, the short-term functional benefits of omega-3 supplementation may be limited, and its clinical relevance warrants further investigation over longer interventions or higher doses.

## Figures and Tables

**Table 1:** Resistance training protocol.

Week	Sets	Repetitions	Intensity (%1-RM)	Rest (s)
1	3	16	50–55	60
2	3	14	55–60	60
3	3	12	60–65	75
4	3	12	65–70	75
5	4	10	70–75	90
6	4	10	70–75	90
7	4	8	75–80	90
8	4	8	75–80	90

1-RM: One-repetition maximum.

**Table 2:** Baseline characteristics of the participants.

	RO	RP	C	P
**Measure**				
**Anthropometry, body composition, health and sleep questionnaires**				
Age (y)	48 ± 3	48 ± 2	49.4 ± 2	0.246
Height (cm)	158.53 ± 5.48	157.60 ± 2.22	158.10 ± 2.07	0.793
Body mass (kg)	72.85 ± 4.73	70.63 ± 1.71	71.06 ± 4.85	0.304
BMI (kg.m^−2^)	29.00 ± 1.64	28.44 ± 0.85	28.42 ± 1.67	0.472
BFP (%)	36.28 ± 2.40	40.59 ± 3.87	39.31 ± 1.53	<0.001
SMM (kg)	29.29 ± 3.80	30.27 ± 3.29	28.38 ± 3.20	0.348
PSQI	2.86 ± 0.91	2.78 ± 0.89	3.13 ± 0.99	0.578
GHQ-28	25.60 ± 6.66	26.64 ± 7.32	27.13 ± 7.89	0.616
**Menopausal status**				
Premenopausal (n)	2	2	0	
Perimenopausal (n)	8	12	9	
Postmenopausal (n)	5	0	6	
**HRT**				
HRT use	3	3	4	
No HRT	12	11	11	
**Performance**				
Bench press (kg)	21.30 ± 3.17	20.92 ± 2.49	20.60 ± 3.22	0.815
Plank (s)	18.22 ± 4.01	19.47 ± 5.1	18.9 ± 4.22	
Leg press (kg)	42.40 ± 5.39	42.50 ± 5.22	41.86 ± 5.20	0.941
Chair stand test (repetitions)	20.53 ± 2.09	21 ± 2.25	20.20 ± 1.61	0.563
Vertical jump (cm)	18.35 ± 2.48	19.96 ± 2.57	18.22 ± 2.57	0.139
Medicine ball throw (m)	4.17 ± 0.42	4.15 ± 0.47	4.21 ± 0.46	0.941
**Biochemical markers**				
GGT (U/L)	36.85 ± 4.81	35.10 ± 5.31	33.92 ± 3.92	0.242
AST (U/L)	24 ± 4.94	23.52 ± 4.08	25.12 ± 4.83	0.636
ALT (U/L)	22.27 ± 3.94	23.73 ± 3.05	23.22 ± 4.11	0.568
BUN (mg/dL)	11.97 ± 2.47	10.88 ± 2.66	11.06 ± 2.81	0.497
Creatinine (mg/dL)	0.67 ± 0.10	0.68 ± 0.14	0.69 ± 0.13	0.859
Follistatin (ng/mL)	1.08 ± 0.18	1 ± 0.17	1 ± 0.14	0.370
Myostatin (ng/mL)	7.40 ± 1.29	7.97 ± 0.95	8.01 ± 0.80	0.221
Follistatin-to-myostatin ratio	0.15 ± 0.04	0.12 ± 0.03	0.12 ± 0.02	0.092

Values are presented as mean ± standard deviation. Abbreviations: PSQI, Pittsburgh Sleep Quality Index; GHQ-28, General Health Questionnaire; BMI, body mass index; BFP, body fat percentage; SMM, skeletal muscle mass; GGT, gamma-glutamyl transferase; AST, aspartate aminotransferase; ALT, alanine aminotransferase; BUN, blood urea nitrogen; HDL, high-density lipoprotein; y, year; cm, centimeter; kg, kilogram; kg.m^−2^, kilogram-meter^−2^; r, repetition; w, watt; deciliter; HRT, hormone replacement therapy; RO, resistance training + 1.6 g·kg^−1^·d^−1^ + omega 3; RP, resistance training + 1.6 g·kg^−1^·d^−1^ + placebo; C, control.

**Table 3:** Mean and standard deviation of macronutrient consumption at pre-test and post-test intervention.

Variable	RO		RP		C	
	Pre	Post	Pre	Post	Pre	Post
**Energy (kcal·kg^−1^·d^−1^)**	25.72 ± 5.13	29.10 ± 2.57	25.37 ± 4.64	29.78 ± 3.39	25.30 ± 6.82	25.31 ± 6.59
**Energy (kcal^−1^·d^−1^)**	1953.73 ± 428.67	2121.18 ± 248.18	1804.61 ± 361.10	2068.31 ± 256.10	1811.22 ± 522	1807.12 ± 527.57
**Carbohydrate (g·kg^−1^·d^−1^)**	3.82 ± 0.71	3.93 ± 0.55	3.99 ± 0.80	3.91 ± 0.56	3.84 ± 1.05	3.85 ± 1.08
**Protein (g·kg^−1^·d^−1^)**	0.59 ± 0.07	1.60 ± 0.009	0.63 ± 0.07	1.59 ± 0.006	0.60 ± 0.06	0.59 ± 0.06
**Fat (g·kg^−1^·d^−1^)**	0.85 ± 0.24	0.85 ± 0.16	0.79 ± 0.26	0.78 ± 0.12	0.83 ± 0.34	0.83 ± 0.29

## Data Availability

The original contributions presented in the study are included in the article, further inquiries can be directed to the corresponding author.
